# Structural Brain Changes Associated with Overweight and Obesity

**DOI:** 10.1155/2021/6613385

**Published:** 2021-07-16

**Authors:** Erick Gómez-Apo, Alejandra Mondragón-Maya, Martina Ferrari-Díaz, Juan Silva-Pereyra

**Affiliations:** ^1^Servicio de Anatomía Patológica, Hospital General de México “Dr. Eduardo Liceaga”, Ciudad de México, Mexico; ^2^Facultad de Estudios Superiores Iztacala, Universidad Nacional Autónoma de México, Tlalnepantla, Mexico

## Abstract

Obesity is a global health problem with a broad set of comorbidities, such as malnutrition, metabolic syndrome, diabetes, systemic hypertension, heart failure, and kidney failure. This review describes recent findings of neuroimaging and two studies of cell density regarding the roles of overnutrition-induced hypothalamic inflammation in neurodegeneration. These studies provided consistent evidence of smaller cortical thickness or reduction in the gray matter volume in people with overweight and obesity; however, the investigated brain regions varied across the studies. In general, bilateral frontal and temporal areas, basal nuclei, and cerebellum are more commonly involved. Mechanisms of volume reduction are unknown, and neuroinflammation caused by obesity is likely to induce neuronal loss. Adipocytes, macrophages of the adipose tissue, and gut dysbiosis in overweight and obese individuals result in the secretion of the cytokines and chemokines that cross the blood-brain barrier and may stimulate microglia, which in turn also release proinflammatory cytokines. This leads to chronic low-grade neuroinflammation and may be an important factor for apoptotic signaling and neuronal death. Additionally, significant microangiopathy observed in rat models may be another important mechanism of induction of apoptosis. Neuroinflammation in neurodegenerative diseases (such as Alzheimer's and Parkinson's diseases) may be similar to that in metabolic diseases induced by malnutrition. Poor cognitive performance, mainly in executive functions, in individuals with obesity is also discussed. This review highlights the neuroinflammatory and neurodegenerative mechanisms linked to obesity and emphasizes the importance of developing effective prevention and treatment intervention strategies for overweight and obese individuals.

## 1. Introduction

Obesity is a major global problem that intimately involves biological, physiological, behavioral, social, environmental, economic, and political factors [1]. Epidemic proportions of obesity have been reached by the end of the century since obesity has become one of the leading causes of death, a worldwide disability [2], and a significant financial burden [3]. In the 1970s, groups of specialists from the United Kingdom and the United States highlighted the need to consider the new phenomenon of obesity as an important disease, which at that time only affected adults, particularly women [4]. Moreover, obesity is exacerbated by the lack of effective treatment options [5].

In 2016, more than 1.9 billion adults aged 18 and older were overweight, of which more than 650 million had obesity. These numbers correspond to 39% of adults over 18 years of age (39% men and 40% women), who were overweight, and 13% of the global adult population (11% men and 15% women) developed obesity [6].

In the last four decades, the prevalence of obesity has nearly tripled worldwide. More than 340 million children and adolescents aged from five to nineteen years were overweight or obese by 2016, and an estimated 38.2 million children under five years of age were overweight or obese in 2019 [6]. If recent trends continue, it is estimated that, by 2030, 60% of the world population (3.3 billion people) could be overweight (2.2 billion) or obese (1.1 billion) [7].

Overweight and obesity are linked to a higher number of deaths worldwide than underweight, with obesity rates surpassing 50% in many countries [8].

Body mass index (BMI) is one of the most widely used measures to identify excessive weight with respect to height and age. The World Health Organization categorizes obesity in terms of BMI: underweight (BMI less than 18.5 kg/m^2^), normal weight (range from 18.5 to 25 kg/m^2^), overweight (range from 26 to 30 kg/m^2^), and obese (greater than 30 kg/m^2^) [6, 9]. However, the use of BMI as an index of overweight or obesity is not reliable for all individuals; BMI has been mainly used in adults, although it is now being used in children and elderly individuals. In the case of children and adolescents, BMI z-scores are used because in this population, BMI varies with age and sex [10] (BMI z-score is defined as an index of relative weight adjusted for child age and sex in relation to a reference population).

The waist-to-hip ratio (WHR) is another widely used measure that indexes the distribution of the adipose tissue [11]. Central body fat is associated with increased deposition of intra-abdominal adipose tissue, although an increase in subcutaneous abdominal adipose tissue is also involved. However, the WHR is not a flawless measure since abdominal circumference alone can provide the same information [11].

Dual-energy X-ray absorptiometry (DXA) is a technique extensively used in the clinic to assess the body composition (bone mineral, fat, and lean and soft tissues) using a low-emission X-ray scan. DXA provides information about excess adiposity and quantifies total fat and lean soft tissue [12]. Air displacement plethysmography (ADP) is another useful technique to assess the body composition (bone density, lean tissue, and total body fat). ADP estimates the body volume of an individual by calculating the volume of the air in an empty chamber minus the volume of the air with a person seated inside the chamber [13].

Skinfolds are a common anthropometric method used to measure subcutaneous fat thickness, although they are not very indicative in some adults with overweight and obesity. Moreover, standardized skinfold caliper measures are lacking; hence, there is no consensus regarding the actual distribution of subcutaneous fat measurements in the population [11].

## 2. Pathogenesis of Obesity

Obesity develops as a consequence of a lack of balance between food intake and energy consumption [14]. Energy expenditure includes the energy required to maintain vital functions (resting metabolic rate), perform physical activity, and provide diet-induced thermogenesis. Published studies have not supported the assumption that obesity is caused by disturbances of energy expenditure related to metabolism and/or diet-induced thermogenesis; instead, evidence suggests that diminished physical activity may significantly contribute to body weight gain [15].

The energy expenditure required to maintain the weight varies widely between individuals, including people with similar constitutions. Differences in metabolic efficiency may explain this variability and play a role in susceptibility to weight gain [15]. Transformations of the lipids and carbohydrates into actual task performance require oxidation of the nutrients to yield adenosine triphosphate (ATP), which serves as a metabolic currency [15], and ATP is subsequently utilized to perform actual tasks (e.g., vital body functions and physical activity) [15]. Both of these metabolic processes involve heat production. Thus, metabolic efficiency refers to the proportion of ATP vs. the production of heat derived for performance of a given task [16]. The ability to dispose a part of excess energy as heat decreases the ability to store excess energy as fat and thus prevents weight gain [16]. Low metabolic efficiency implies an increase in heat production at the expense of ATP production [16]. Enhanced metabolic efficiency has been reported to contribute to obesity [16].

The sympathetic nervous system (SNS) participates in homeostatic control. Fasting reduces SNS activity, while eating, especially carbohydrate overfeeding, increases SNS activity. The SNS innervates and modulates lipolysis in the adipose tissue [17]. Parasympathetic input may mediate the etiology of obesity by directly influencing the metabolic state of the adipose tissue. Neuroimmune interactions between the SNS and macrophages are required for homeostasis of multiple tissues, including the adipose tissue [18]. Thus, SNS-mediated reduction in fat tissue lipolysis contributes to lipid accumulation and, consequently, excess weight [17].

The brain and gastrointestinal tract are connected via the vagus nerve. Intra-abdominal adipocytes promote glucose and fatty acid intake by stimulating the SNS; these cells express adrenergic receptors that can respond to catecholamines of the sympathetic system. The cholinergic part of the vagus nerve participates in the regulation of glucose and insulin [17]. Acetylcholine acts through the vagus nerve to potentiate the M3 muscarinic receptors of the pancreas to increase insulin secretion, improving the synthesis of cellular lipids and absorption of glucose, which cause storage of the calories and gain of lipids [17]. The enteric nervous system produces more than 30 neurotransmitters; these hormones and peptides are released into the bloodstream, cross the blood-brain barrier (BBB), and stimulate the central nervous system (CNS). During ingestion, intestinal hormones are released due to the distension of the stomach, such as the peptides cholecystokinin, ghrelin, and leptin, which regulate the sensations of hunger and satiety. Ghrelin stimulates food intake by inhibiting the vagal signals and suppressing the release of insulin [17].

Leptin and insulin are involved in these effects on SNS activity. Leptin is an adipocyte-produced hormone that is upregulated in obesity [19]. Leptin signals in the brain largely at the level of the hypothalamus to modulate the activity of specific neuronal subsets (including orexigenic Agouti-related peptide (AgRP) and anorexigenic proopiomelanocortin (POMC) neurons), reduce appetite, and increase energy expenditure [20]. Leptin functions as a signal to circulate energy reserves by providing feedback inhibition in the hypothalamic orexigenic pathway; thus, obesity is strongly associated with hyperleptinemia [5]. Obesity is characterized by impaired leptin signaling despite elevated leptin levels, that is, leptin resistance, which explains why leptin administration to most people with obesity is not effective [20]. Leptin resistance is believed to be a result of inflammation and hypothalamic gliosis [20–22]. Long-term feeding of a high-fat diet to rats increases mediobasal hypothalamic activation of the inflammatory signaling intermediates c-Jun N-terminal kinase (Jnk) and nuclear factor-*κ*B (NF-*κ*B), which results in the production of proinflammatory cytokines and impairment in insulin and leptin signaling [22]. Additionally, cellular response to a high-fat diet in the hypothalamus involves reactive gliosis [18], which is a CNS-specific process of recruitment, proliferation, and morphological transformation of astrocytes and microglia in response to brain injury. This cellular response alters neurovascular coupling of POMC neurons due to ensheathment of the synapses to modify neurotransmitter dynamics by altering astrocyte expression of glutamate and glucose transporters to change the firing activity of POMC neurons [22].

Complex systems that regulate the eating behavior balance the relationships between intake (diet) and energy expenditure. These systems are vulnerable to the disturbances caused by energy imbalance. The hedonic and homeostatic pathways control the eating behavior. The hedonic system relies on the striatum and has close connections to the hypothalamus and homeostatic system [23]. A reward deficiency is considered to cause an imbalance between homeostatic and hedonic regulation. This hypothesis suggests that decreased dopaminergic signaling, which typically transmits the rewarding aspects of (food-related) stimuli, promotes overconsumption of tasty foods beyond homeostatic needs to compensate for lower sensitivity to the reward [24]. Decreased serotonin signaling in the hypothalamus is assumed to contribute to obesity by affecting negative feedback of energy ingested at food intake, thereby promoting excessive consumption [25].

The paraventricular nucleus of the hypothalamus produces peptides that decrease with food intake, including thyrotropin-releasing hormone, corticotropin-releasing factor, and oxytocin. Cannabinoids regulate appetite, and inflammatory cytokines are also involved in the regulation of the gastrointestinal nervous system [19]. Inflammation in the hypothalamus can produce widely variable effects on behavior. Thus, substantial elevations in hypothalamic cytokine levels have been observed in deep-anorexia animal models, both dependently and independently of leptin [5]. Hypothalamic resistance to the effects of leptin on the adipose tissue is essential for obesity. Hypothalamic inflammation is triggered in response to the consumption of large amounts of fat on a daily basis and is an important mechanism in the development of leptin resistance [26].

de Araujo et al. [27] considered the important effects of the energy content of food mediated by gut-innervating sensory vagal neurons (the gut-brain axis pathway) that apparently acts as a form of an interoceptive reward system, which is independent of palatability. Individuals with obesity do not report liking food more than their healthy-weight counterparts. Palatability affects what an individual eats but does not reflect how much this individual eats. The energy content of food is reinforcing. According to de Araujo et al. [27], subcortical gut-brain pathways sense nutritive properties independently of palatability and activate the brain reward circuits. The motivational power of energy density appears stronger in people with obesity.

However, the gut microbiota is thought to play a role in the mechanisms governing the stress response via the hypothalamic pituitary adrenal (HPA) axis, and a deregulation of this axis has also been related to obesity [28]. The gut-brain axis is a complex system that enables communication between the gut and brain through hormonal, immunological, and neural signals. The CNS, autonomous nervous system, enteric nervous system, HPA axis, and gut microbiota are components of the gut-brain axis. All these components establish bidirectional communications from the CNS to the gut and from the gut to the CNS [29].

Gut hormones are released by enteroendocrine cells that trigger signaling in the CNS in response to preabsorptive nutrients and subsequently impact energy homeostasis [29]. As a part of the gut-brain axis, the gut microbiota (microorganisms that live in the human digestive tract) play an important role in adiposity and weight gain via energy harvesting, posterior generation of various metabolites (e.g., short-chain fatty acids), changes in host behavior, and satiety through the gut-brain axis that induces inflammatory responses [29]. Obesity, metabolic diseases, some psychiatric disorders, and cognitive impairments can result from dysregulation of this system [28, 29].

There are positive associations between stress (high levels of glucocorticoids), weight gain, adiposity, BMI [30], basal glucose, basal insulin, and resistance to insulin [31]. Associations of stress with metabolic dysfunction are stronger in individuals with higher BMI than that in people with lower BMI [32], suggesting that stress increases obesity risk, especially in individuals with higher BMI. Chronic high levels of glucocorticoids and insulin boost an increase in palatable food intake and abdominal fat deposition [33]. Stress can trigger metabolic dysfunction and modify eating behavior; moreover, individuals with obesity are more sensitive to stress. The HPA axis plays an important role in the onset of metabolic alterations and obesity [34].

## 3. Obesity and Neuroinflammation

Macrophages regulate inflammation according to various activation states depending on their state of differentiation; thus, classically activated macrophages (M1) initiate the process by secreting proinflammatory cytokines and reactive oxygen species (ROS) [35], whereas subsequent phases of the immune response are controlled by “alternatively” activated macrophages (M2) to reduce inflammation and promote tissue remodeling and release of the growth factors [35].

In healthy, nonobese humans, macrophages in the adipose tissue apparently function similar to M2 macrophages; that is, they produce little to no proinflammatory cytokines and express arginase, which inhibits nitric oxide production and leads to polyamine generation [36]. However, adipose-tissue-associated macrophages located at the sites of chronic inflammation in obesity can act as the sources of proinflammatory cytokines [37]. Both M1 and M2 macrophages may coexist, leading to persistent inflammation and fibrosis [38].

Accumulation of adipose tissue in obesity is the key factor of systemic inflammation. Both hypertrophic adipocytes and immune cells residing in the adipose tissue (mainly lymphocytes and macrophages) contribute to a proinflammatory state via an increase in the levels of tumor necrosis factor alpha (TNF-*α*), plasminogen activator inhibitor-1, C-reactive protein, interleukin-1-beta (IL-1-beta), and interleukin-6 (IL-6) [39, 40]. The inflammatory response mediated by M1 during obesity may be analogous to the mechanisms of necrotic clearance [41, 42]. Macrophages secrete cytokines, especially TNF and IL-6, and chemokines, such as CC-chemokine ligand 2 (CCL2; previously known as monocyte chemotactic protein-1 (MCP1)). TNF and IL-6 can interfere with insulin signaling in adipocytes, leading to type 2 diabetes (T2DM) [43]. Macrophages accumulate in adipose tissue over time, and cytokines that they produce can lead to insulin resistance and T2DM [36, 37]. Overexpression of procoagulant proteins by these inflammatory macrophages can contribute to atherogenic and cardiovascular risks that constitute a part of obesity-associated metabolic syndrome [43].

However, obesity-associated gut microbiota may also contribute to neurochemical and inflammatory alterations [28, 44]. The gut microbiota is an intermediate factor between environmental pressures (e.g., diet and lifestyle) and host physiology, and its alteration (i.e., dysbiosis) can partly explain obesity [28]. Gut dysbiosis (imbalance in gut microbiota composition caused by host genetics, lifestyle, and exposure to microorganisms) [45] may promote diet-induced obesity and metabolic complications via a variety of mechanisms, including immune dysregulation, altered energy regulation, altered gut hormone regulation, and proinflammatory mechanisms (such as lipopolysaccharide endotoxins that cross the gut barrier and enter the portal circulation) [44, 46]. Recent studies demonstrated that compositional changes in the gut and inflammation related to a leaky gut (a loss of intestinal barrier integrity reducing its ability to protect the internal environment) may contribute to the pathophysiology of several diseases, such as depression, chronic fatigue syndrome, obesity, or T2DM [47].

Inflammation resulting from obesity can affect brain structures, such as the hippocampus, cerebral cortex, brain stem, and amygdala [48]. Low-grade inflammation characteristic for obesity can lead to neuroinflammation via various mechanisms, including the choroid plexuses and disruption of the BBB [49]. Peripheral inflammation observed in obesity leads to insulin resistance [36, 37]. The brain is a privileged organ for immunity; however, transitions between peripheral and central inflammation have been reported. Adipokines are produced by the adipose tissue and can also be expressed in the CNS, where receptors for these factors are present. Adipokines produced in the periphery can cross the BBB or modify its physiology by acting on the cells that form the BBB to affect the CNS. Adipokines can regulate neuroinflammation and oxidative stress, which are two important physiological processes involved in neurodegeneration and are associated with many chronic neurodegenerative diseases [50]. Damage to the BBB in aging can also lead to inflammation in the brain. Neuroinflammation may be the most important cause of cognitive dysfunction and can thus lead to a central pathological mechanism associated with aging [51].

Table 1 lists the cytokines and chemokines induced by various cell types and activation states of the cells (either quiescent or activated) in fatty tissue outside the CNS and their relations to microglia. Evidence supporting the involvement of two recently recognized factors linking obesity to neurodegeneration is based on the induction of proinflammatory cytokines and resistance to insulin-like growth factor 1 (IGF-1) [39, 40, 50–52]. Central inflammation in obesity leads to interruption of hypothalamic satiety signals and perpetuation of overeating and also to negative results in cognition [53]. Chronic inflammation is considered a part of the pathogenic mechanisms of diverse disorders linked to aging. Peripheral inflammation and related metabolic disturbances facilitate not only insulin resistance and T2DM but also neurodegenerative disorders [54].

**Figure 1:**
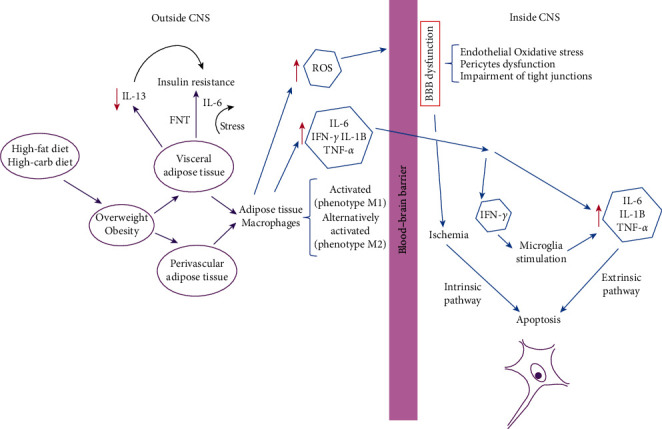
Pathogenesis of obesity-associated brain damage. Outside CNS: adipose tissue macrophages infiltrate the adipose tissue in obesity and contribute to insulin resistance. M1 or “classically activated” macrophages are induced by proinflammatory mediators, such as lipopolysaccharides and IFN-*γ*. M1 macrophages enhance proinflammatory cytokine production (TNF-*α*, IL-6, and IL-12), which can block the effects of insulin action on adipocytes (link between inflammation and insulin resistance) and generate reactive oxygen species (ROS), such as nitric oxide, via activation of iNOS (Nos2). Inside CNS: local and systemic inflammation induced by obesity can cause breakdown of the blood-brain barrier (BBB), a decrease in waste removal, and an increase in infiltration of immune cells. Microglia: activated microglia secrete proinflammatory cytokines (IL-1*β*, IL-6, TNF-*α*, and INF-*γ*), which stimulate inflammatory signaling in adjacent neurons, which in turn induces insulin and leptin resistance of neurons.

Macrophages in the adipose tissue of the abdomen promote an increase in cytokines and proinflammatory chemokines that can cross the BBB. Interferon-gamma can activate microglia that act as a relay for neuroinflammation [62]. Hypertension, diabetes, and obesity synergistically promote various pathological mechanisms, including cerebral hypoperfusion and glucose hypometabolism. These risk factors trigger neuroinflammation and oxidative-nitrosative stress. Proinflammatory cytokines, endothelin-1, and oxidative-nitrosative stress cause several cycles of pathological feedback and influence cerebrovascular pathology [63]. These cascades lead to an increase in neuronal Ca^2+^ and neurodegeneration [64]. Oxidative-nitrosative stress promotes long-term damage that involves fatty acids, proteins, DNA, and mitochondria. These factors amplify and perpetuate various pathological feedback loops [65]. Dysfunctional energy metabolism (compromised production of mitochondrial ATP), generation of *β*-amyloid, endothelial dysfunction, and alteration of the BBB [39, 51] lead to a decrease in the cerebral blood flow and chronic cerebral hypoperfusion. Thus, hypoperfusion deprives the brain of its two most important trophic substances, oxygen, and nutrients. Consequently, the brain suffers from synaptic dysfunction and neuronal degeneration or loss, leading to gray and white matter atrophy [65] (see Figure 1).

The sources of inflammation in many neurodegenerative diseases have been linked to a decrease in the M2 macrophage population in the CNS and a subsequent increase in M1-mediated neuroinflammation [62]. Macrophage-stimulating protein receptor (MST1R) is a receptor tyrosine kinase expressed in tissue-resident macrophages, including microglia. MST1R activation by its ligand, a macrophage-stimulating protein, attenuates obesity-mediated inflammation in the periphery. In vivo cleavage to the MST1R ligand promotes inflammation (M1) and limits the activation of macrophage-dependent repair (M2) [66].

Neuroinflammation can lead to apoptosis [67]. This basic physiological mechanism is a highly regulated process of cell death that contributes to the maintenance of homeostasis. A lack of regulation of apoptosis plays a key role in the etiology and/or progression of many diseases [67, 68]. Complete implementation of apoptosis involves interplay between a wide array of proteins, signal transducers, and signaling pathway cascades [69]. TNF, Fas ligand (Fas-L), and TNF-related apoptosis-inducing ligand (TRAIL) bound to the extracellular domain of DR (transmembrane receptors) are responsible for the initiation of the major apoptosis pathway, that is, the TNF pathway [67–69]. TNF-*α* and Fas-L can induce apoptosis of some neurons during inflammation [67]. Apoptosis involves the activation of caspases (cysteine proteases), which orchestrate all morphological changes that characterize this form of cell death [67]. Activation of initiator caspases (caspase 2, 8, or 10) is the first step in a regulated, irreversible, and self-amplifying proteolytic pathway that leads to the activation of effector caspases (caspases 3, 6, and 7) [70].

Inflammation on its own causes hypoperfusion and ischemia [41], which can lead to apoptosis via the intrinsic route [68]. This pathway is characterized by nonreceptor-mediated initiation and mitochondrial regulation. In the intrinsic pathway of apoptosis, certain stimuli directly generate intracellular signals that lead to biochemical changes within the cells [70]. A stimulus induces a disruption of the mitochondrial membrane that dissipates the membrane potential, enhancing membrane permeability [70]. The stimulus also results in the formation of the mitochondrial permeability transition pore (MPT) on the outer membrane that channels proapoptotic factors into the cytosol [71]. Apoptosomes cleave procaspase-9 to yield active caspase-9, which in turn activates the effector caspase (i.e., caspase-3) [70].

## 4. Macroscopic Brain Changes (Neuroimaging Study Evidence)

### 4.1. Gray Matter

A recent systematic review [72] revealed structural abnormalities in the gray matter volume in patients with obesity. Analysis of ten studies up to December 2017 [73–82] indicated a consistent reduction in gray matter in individuals with obesity linked to the control regions in the inferior frontal gyri, right insula, left and right precentral gyri, left middle frontal gyrus, left middle temporal gyrus, left amygdala, and left cerebellar hemisphere. However, the analyzed studies demonstrated an increase in the volume of gray matter in the left cuneus, left middle frontal gyrus, and left inferior occipital gyrus. Similarly, Kurth et al. [83] reported negative correlations between BMI and the volume of the left superior frontal gyrus, left middle and inferior frontal gyri, right frontal pole, left insula, and bilateral superior and middle temporal gyri. A neuroimaging study in 1,428 participants [84] detected that the volume of gray matter negatively correlated with BMI in the right inferior frontal gyrus, superior frontal gyri, right precentral lobe, right superior parietal lobule, left and right precuneus, left fusiform gyrus, left and right uncus, left and right anterior lobes of the cerebellum, and right brain stem in men. BMI positively correlated with the volume of some regions of the inferior frontal gyri, right superior frontal gyrus, and left and right posterior lobes of the cerebellum. Higher BMI in older adults is associated with generalized gray matter disturbances, regardless of obesity-related comorbidities and other confounding factors, which translate into subtle disturbances in memory performance in aging [85]. García-García et al. [86] reported that obesity and body mass are related to significantly lower gray matter volume in the brain areas with a key role in executive control. Variables related to obesity are consistently associated with a reduction in the gray matter volume in various areas, including the medial prefrontal cortex, bilateral cerebellum, and left temporal pole. Similarly, individuals with obesity have a lower total gray matter volume than lean and overweight individuals. In adolescents, BMI is related to global and regional differences.

Yokum et al. [87] detected a lower volume of gray matter in the regions involved in inhibitory control, which is related to future weight gain. Specifically, abnormalities in the regional gray matter volumes, but not in white matter volumes, increase the risk of future weight gain, and abnormalities in the regional white matter volumes, but not in the gray matter volumes, are secondary to weight gain.

### 4.2. White Matter

There is a strong evidence of a global white matter reduction associated in subjects with elevated BMI [88, 89]. This relationship is consistent with a large-scale study that demonstrated associations between elevated BMI and reduced white matter integrity in two large independent cohorts [90]. A higher BMI is associated with a decrease in various white matter regions, including anterior and posterior thalamic radiations, inferior fronto-occipital fascicle, inferior and superior longitudinal fascicles, corpus callosum [72], uncinate fascicle, internal capsule, corticospinal tract, and cingulum (cingulate gyrus and hippocampus) [76, 79, 88–91]. Local changes in the white matter fiber tracts associated with increased BMI link the key limbic structures with prefrontal regions, possibly explaining increased risk for cognitive impairments and dementia in obesity in older age [64]. Alterations mainly include fiber tracts that link limbic structures with prefrontal regions and may promote accelerated aging in people with obesity, which is expected to increase the risk of cognitive impairment [91].

BMI-related WM changes are associated with microstructural changes in white matter. The results of voxel-based quantification analysis indicated a reduction in white matter myelin, increase in the water content, and alterations in the iron content in subjects with increasing BMI, which are predominantly localized in the superior longitudinal fasciculus (SLF), anterior thalamic radiation (ATR), internal capsule, and corpus callosum [91]. Increased BMI is indicative of a loss of white matter integrity via demyelination or inflammatory effects. These changes can be explained by axonal damage or cellular loss [91].

### 4.3. Subcortical Areas

The sizes of the bilateral thalamus, putamen, and globus pallidus are enlarged, and the size of the bilateral caudate is reduced in individuals with obesity compared with those in individuals with normal weight [92]. The medial-dorsal part of the bilateral caudate is significantly reduced, and the lateral dorsal part of the bilateral thalamus is significantly increased. Even overweight individuals (with a BMI of 25–30 kg/m^2^) have signs of atrophy in the basal ganglia and radiated crown [93]. In men, total body fat is negatively correlated with all subcortical gray matter volumes (thalamus, caudate nucleus, putamen, globus pallidus, and nucleus accumbens); in women, a greater total body fat is correlated with lower globus pallidus volume [94]. These sex-specific differences may be explained by several endocrine pathways; for instance, estrogens act as a protective factor against many metabolic complications related to obesity [95].

Kim et al. [92] suggest that, in individuals with obesity, subcortical structures are associated with feeding behavior and sensory function. Higher BMI is associated with greater hypothalamic connectivity in the regions involved in food motivation and reduced connectivity with the structures associated with cognitive control of food intake. The hypothalamic gray matter volume is positively correlated with connectivity of the posterior insula of the hypothalamus. Thus, the relationships between the hypothalamic gray matter volume and BMI are mediated by connectivity of the hypothalamus-posterior insula [96].

### 4.4. Children and Adolescents

Compared with children with a normal weight, children with obesity manifest a significant reduction in gray matter in the right medial temporal gyrus, right and left thalamus, left superior parietal gyrus, left pre- and postcentral gyri, and left cerebellum [97]. Childhood obesity is apparently associated with higher structural volumes and decreased gray matter density in the reward system [98]. Additionally, there are some differences in white matter between children with obesity and children with a normal weight, and excess body fat has been associated with differences in structural connectivity, particularly in the frontal and temporal brain regions [97].

Notably, adolescence is a critical period of brain development, particularly the maturation of the prefrontal cortex, which is an area related to cognitive control. In adolescence, less developed prefrontal cortex may explain lower capacity to regulate reward-driven behaviors, such as consumption of calorie-dense foods. This overconsumption of calorie-dense foods can subsequently affect functional and structural maturation of the brain to consequently influence cognition and behavior [99]. However, how obesity affects brain developmental trajectories (possible early maturation of appetite regulatory brain regions) is poorly understood; therefore, longitudinal studies are required.

Sharkey et al. [100] did not detect significant associations between cortical thickness and BMI. The results remained negative when analysis was restricted to children from 12 to 18 years of age. Therefore, associations between BMI and cortical thinning appear to develop after adolescence. This finding has implications for the nature of the relationships between brain structure and weight gain. In this context, adolescents who gained body fat manifested a greater decrease in the gray matter volume in the putamen compared with those who lost body fat. Moreover, the former subjects manifested a greater increase in the white matter volume in the anterior cingulate cortex compared with the latter subjects and subjects who maintained a stable level of body fat [101].

Overweight and obese adolescents manifested diminished white matter integrity in the superior frontal corpus callosum, bilateral uncinate fasciculi, left inferior fronto-occipital fasciculus, and left corticospinal tract compared to adolescents with a normal weight. These findings may be associated with an increase in the reward system [102]. However, overweight and obese adolescents manifested heightened white matter integrity in the orbital and anterior frontal corpus callosum, right inferior fronto-occipital fasciculus, left cingulum, and left corticospinal tract compared to adolescents with a normal weight. This result may be associated with reinforced attentional processing [102].

### 4.5. Allostatic Load

Individuals who are overweight had a lower cortical thickness in the left and right superior frontal gyrus than the control subjects [103, 104]. Furthermore, these subjects had higher rates of allostatic loading than the control or lean individuals. The allostatic load index comprises 15 biomarkers: systolic and diastolic blood pressure, glycated hemoglobin, glucose, creatinine, total cholesterol, HDL and LDL cholesterol, triglycerides, C-reactive protein, interleukin-6, insulin increase, cortisol, fibrinogen, and leptin. An increase in the allostatic load is related to the changes in the composition of the gray matter in the regions that control eating behavior, sensory reward processing, and general cognitive function [104]. A reduction in gray matter reduction has been suggested to be related to a decrease in creatinine and an increase in triglycerides and low-density lipoproteins in the plasma of subjects with obesity [105].

### 4.6. Metabolic Syndrome

A metabolic profile characterized by high body fat content, visceral adiposity, and systemic inflammation is associated with reduced gray matter volume and reduced executive function in older adults [106]. Factors underlying sustained energy imbalance, such as sedentary lifestyle or high-energy food intake, can be important determinants of the changes in gray matter in aging. Associations between metabolic syndrome and decreased gray matter volume have been detected in multiple brain regions, including the posterior cerebellum, brain stem, orbitofrontal cortex, bilateral caudate nucleus, right parahippocampus, right amygdala, right insula, lingual gyrus, and right superior temporal gyrus [107].

### 4.7. T2DM

T2DM is associated with a decrease in total gray matter volume and hippocampal volume, increase in WHR and BMI, and lower physical activity [108]. Adolescents with T2DM and obesity have reduced gray matter volume in the right hippocampus, left putamen and caudate, bilateral amygdala, and left thalamus compared with controls with a normal weight [109]. Abdominal obesity or its subsequent effects may partially mediate the adverse effect of T2DM on brain atrophy [108]. Adolescent obesity alone appears to result in a reduction in gray matter volume, and T2DM is associated with abnormalities in the white and gray matter in this population [109]. Structural alterations in parietal and occipital cortices may constitute early cerebral alterations related to T2DM [110]. T2DM is associated with significant regional changes in the fiber density within the corpus callosum, fornix, left inferior fronto-occipital fascicle, left uncinate, and left internal and external capsules [109, 111].

Comparison with control subjects indicated that patients with obesity and T2DM have lower thickness of the right insula and left lateral occipital [110] and temporal [112] cortices. The global mean cortical thickness was lower in the T2DM with obesity group than that in the group of T2DM individuals with a normal weight [113]. A negative correlation between disease duration and mean overall white matter integrity has been reported in individuals with T2DM and obesity, but not in the T2DM subjects with a normal weight [113]. Normoglycemic patients with obesity manifested reduced thickness at the right insula and inferior frontal gyrus and greater amygdala and thalamus volumes than the control subjects. In contrast, the volumes of the thalamus and left paracentral area were greater in the normoglycemic group compared with those in patients with obesity and T2DM [110].

### 4.8. Other Anthropometric Variables

In addition to BMI, WHR and waist circumference are also associated with cortical thickness. Higher WHR and greater waist circumference are associated with a lower volume of gray matter [83, 114–116]. Hayakawa et al. [116] detected that a higher number of brain regions in women correlated with waist circumference than with BMI. The highest levels of all obesity measures (i.e., BMI, WHR, and fat mass) were related to lower gray matter volume. The combination of general and central obesity (WHR> 0.85 for women and >0.90 for men) is associated with a lower gray matter volume than that in lean adults [117]. However, the body composition, which distinguishes between fat mass and fat-free mass, is not directly associated with changes in the brain tissue volume and white matter integrity. Cross-sectional associations between the body composition and brain tissue volume probably reflect a cumulative risk or a shared etiology [118].

Kurth et al. [83] demonstrated negative correlations between both BMI and waist circumference and the gray matter volume, and the regional effects of the gray matter are greater in the hypothalamus and include the parietal, prefrontal, anterior temporal, and inferior cortices and the cerebellum. Interestingly, the associations were more generalized and pronounced in the case of waist circumference than in the case of BMI. These relationships significantly differ depending on sex. The associations with both BMI and waist circumference are similar in men; however, women presented with a higher number of correlations with waist circumference. Kurth et al. [83] suggested that waist circumference is a more sensitive indicator than BMI, particularly in women, to potentially determine the adverse effects of obesity and overweight on the brain.

Overall, there is evidence of extrahypothalamic damage, with a decrease in cerebral cortical volume predominantly in the frontal and temporal lobes. Furthermore, obesity has been clinically related to cognitive deficits.  Table 2 presents a summary of the findings of the neuroimaging studies. The relationships between a decrease in the thickness of the cerebral cortex and cognitive deficit suggest a microscopic disturbance in neuronal tissue. Neuronal loss may be due to the activation of apoptosis through the intrinsic pathway due to mitochondrial damage or the activation of the extrinsic pathway due to neuroinflammation. Furthermore, cognitive deficits may be associated with altered synapses and a lack of pruning of dendritic spines due to poor microglial function.

## 5. Microscopic Brain Changes

One of the initial reports on the adverse effects of experimental obesity in brain pathology described the alterations in the lipid composition of myelin in genetically obese mice [119]. Several pathophysiological processes are related to high adiposity, including endothelial dysfunction and inflammation [120]. Neuronal loss appears to be accelerated by several factors that increase ischemia [9]. Other studies have shown that obesity in healthy mature adults is associated with axonal and/or myelin abnormalities in the white matter and decreased gray matter volumes that can reflect neuronal loss [119].

In humans, histological evidence is very limited and contradictory to what has been observed in the imaging studies [121]. A recent postmortem stereological study investigated the numbers of the cells in the striated nucleus in subjects with obesity. This study is one of a few that analyzed postmortem brain tissue in the context of obesity. Cellular base and parenchyma in postmortem brain tissue were investigated by a combination of histochemical and immunohistochemical methods to quantify the total numbers of neurons and astrocytes in the striated nuclei of nine obese subjects (BMI 40.2 kg/m^2^) and eight controls (BMI 24.4 kg/m^2^). No differences in the average total number of neurons or astrocytes were detected; however, the variance in the number of neurons, but not astrocytes, was increased in the group with obesity [122].

Recently, Gómez-Apo et al. [123] carried out a study in Mexico to compare cortical thickness and neuronal cell density in the postmortem brain tissue of eight donors who were overweight or obese (mean BMI 31.6 kg/m^2^; SD = 4.35; *n* = 8; 6 men) and eight donors with normal weight (mean BMI 21.8 kg/m^2^; SD = 1.5; *n* = 8; 5 men). Coronal sections were processed to obtain high-resolution images of real size, and the thickness of the three frontal and temporal gyri of each hemisphere was measured. The numbers of neurons were also quantified in 64 randomly selected fields of the first, second, and third bilateral frontal and temporal gyri. Although significant differences in the cortical thickness were not detected in the groups, significantly fewer neurons were present in the overweight/obesity group than that in normal weight individuals in various frontal and temporal areas.

## 6. Discussion and Conclusion

Excess visceral fat is accompanied by increased circulating levels of proinflammatory cytokines and acute phase reactants, which lead to mild chronic inflammation that affects the liver, adipose tissue, skeletal musculature, and vasculature [5, 124]. Obesity is associated with damage to various tissues and is related to functional and structural changes in the brain [78]. A long-term high-fat diet reduces the number of synapses on hypothalamic neurons and increases neural apoptosis [125]. The exact mechanisms of the effects of obesity on the development of these disorders are complex and multifactorial, despite being of substantial relevance.

Generally, neuroimaging evidence suggests that obesity is associated with structural abnormalities of the brain. However, some inconsistencies are related to the cortical brain regions where certain reductions and increases have been detected in subjects with obesity. These inconsistencies can be explained by heterogeneity of the patient population, need for large-scale studies, differences in analysis methodology, selection of covariates used in statistical model [126], and wide variety of processing software and the size of smoothing kernel.

A reduction in the gray matter volume has been mainly observed in the cerebellum, basal nuclei, left and right inferior frontal gyri, bilateral superior frontal gyrus, superior and middle temporal gyri, and precentral gyrus. A reduction in volume has also been observed in the white matter, specifically in the corpus callosum, posterior and anterior thalamic radiations, internal and external capsules, inferior and superior longitudinal fascicles, inferior fronto-occipital fasciculus, uncinate fascicle, and cingulum [72, 90]. These fascicles are the most extensively studied structures and are apparently involved in cognitive functions [127].

Obesity is an important risk factor for the onset and progression of various neurodegenerative diseases. Parkinson's disease, Alzheimer's disease, and multiple sclerosis can be initiated by various metabolic changes related to the damage of the CNS caused by obesity [128]. These changes can alter synaptic plasticity of neurons and lead to neuronal death, affecting normal physiology of the CNS [49, 129]. For instance, insulin resistance may be associated with Alzheimer's disease and mild cognitive decline [130]. These associations may be due to the associations of brain aging with reduced insulin effectiveness and inadequate cellular response to insulin or insulin deficiency attributable to reduced transport through the BBB. Moreover, being overweight predisposes individuals to reduced brain volume, brain atrophy, or decreased density of gray matter in the temporal lobe, which may also increase vulnerability to the development of Alzheimer's disease in the future [9, 93].

Obesity-related structural changes in the brain can explain poor cognitive performance of overweight and obese individuals [53, 85, 106]. Beyer et al. [106] demonstrated that high body fat, visceral adiposity, and systemic inflammation are associated with reduced gray matter volume and executive functions. This result expands previous findings of reduced executive function related to increased BMI and reduced gray matter volume in distributed brain regions mediating this effect [85]. Significantly poor performance has been observed in cognitive domains, such as memory (short-term memory and learning problems), speed and psychomotor coordination, verbal fluency, and attention in people with obesity compared with subjects with a normal weight [40, 131–135]. A recent systematic review by Favieri et al. [136] presented consistent results on obesity and disturbances in three cognitive processes considered executive functions: cognitive flexibility, inhibition, and working memory. Cognitive flexibility, which is the ability to change plans or strategies, appears to be more profoundly affected in individuals with obesity than in overweight individuals or individuals with a normal weight [136]. Moreover, longitudinal studies have shown that cognitive flexibility can predict weight loss, in addition to other cognitive and motor variables [137, 138]. Inhibition and working memory are negatively associated with weight and BMI. Both functions seem to predict weight loss and, in turn, have improved after weight loss [136].

Over time, the conditions associated with obesity (e.g., hypertension, diabetes, and apnea) favor pathophysiological changes (e.g., adipokine alterations, insulin resistance, inflammation, and endothelial dysfunction). These changes can be associated with brain disturbances, such as frontal metabolic and circulatory dysfunctions, changes in white matter, and atrophy of the frontal and temporal regions. Brain dysfunction will induce cognitive deficits described in this population (e.g., memory, processing speed, attention, and executive functions) [134].

Specific behavioral patterns have been identified in obese individuals, including the lack of initiative, inhibition, and self-monitoring, which can be expressed as apathy, impulsivity, and poor behavioral control. Interactions between cognitive deficits and behavioral patterns can account for failure to acquire and maintain healthy habits (e.g., balanced diet, physical activity, and exercise). Therefore, cognitive and behavioral patterns of these individuals appear to stimulate weight gain and prevent the use of effective strategies to lose weight.

Obesity has also been associated with a variety of vascular pathologies potentially related to atrophy, including thickening of the carotid artery wall, vascular and coronary endothelial dysfunction, peripheral resistance, arterial stiffness, and ventricular hypertrophy [9]. The pathogenesis of brain impairment caused by chronic hyperglycemia is complex and includes mitochondrial dysfunction, neuroinflammation, impaired neurotransmitters, and vascular disease, leading to cognitive decline, neurodegeneration, and loss of synaptic plasticity.

Neurodegenerative and ischemic injuries exacerbate each other, leading to more serious consequences for cognition than any pathology alone. Examples of such component-specific pathogenic pathways include central insulin resistance and hypoglycemia in diabetes, neuroinflammation, and adipokine imbalance in obesity and atherosclerosis and lipohyalinosis in subjects with high blood pressure [139]. Today, it is evident that subtle but continuous neuroinflammation may provide a foundation for certain disorders, such as small vessel brain disease. Furthermore, obesity, hypertension, diabetes, and atherosclerosis may act as “silent contributors” to promote a chronic proinflammatory state. This state may aggravate the outcome of various pathological processes and contribute to a series of subsequent complications, such as stroke and neurodegeneration, creating a vicious pathological circle [140].

Animal studies using Zucker obese rats (a rat obesity model) demonstrated the presence of diabetes and moderate systemic arterial hypertension. These rats have concurrent hyperglycemia, hyperinsulinemia, and hyperlipidemia. This model was able to demonstrate vascular alterations in the BBB and endothelium compared with nonobese rats. A decrease in the luminal diameter and an increase in vascular wall thickness were detected in the intracerebral arteries of older obese rats [141]. Additionally, the role of megalin, which is a component of the BBB, in the transport of relevant hormones to the CNS has been studied in rodents. The roles of this protein in the brain endothelium and leptin signaling were investigated using a megalin-deficient mouse model specific for endothelial cells. Megalin expression in the brain endothelium was shown to be related to metabolic changes mediated by the leptin signaling pathway, leading to an assumption that obesity is potentially associated with neurodegenerative changes [142].

In conclusion, overweight and obesity generate a series of physiological changes, such as low-grade chronic inflammation (release of cytokines and chemokines that reach the CNS), insulin resistance (increases in ROS), and microangiopathy (i.e., vascular damage of small vessels), which promote neuronal loss. Thus, lower cortical thickness (mainly in the cerebellum and frontal and temporal regions) was detected in overweight and obese individuals. This evidence may explain poor cognitive performance and maladaptive behavior pattern that characterize individuals with overweight and obesity.

## Figures and Tables

**Table 1:** Cytokines and chemokines outside CNS and in relation with microglia.

References	Cell	Interleukins and chemokines
Makki et al. [55]	Quiescent adipocyte	Adiponectin
		Transforming growth factor *β* (TGFβ)
		IL-10
		IL-4
		IL-13
		IL-1 (receptor antagonist)
		Apelin
	Activated adipocyte (obesity)	TNF-α
		IL-6
		Leptin
		Adiponectin
		Resistin
		Visfatin
		Angiotensin II
		Plasminogen activator inhibitor-1 (PAI-1), also known as endothelial plasminogen activator inhibitor)

Wentworth et al. [56]	Adipose tissue macrophages (lean)	Mannose receptor (CD206^+^)
Esser et al. [57]		IL-10
Wang et al. [58]		Arginase (ARG1)
	M1 macrophages (proinflammatory activity)	Platelet-derived growth factor *β* (PDGF-*β*)
		Caspase 1 activation (inflammasomes: CD206^+^ / CD11c^+^)
		IL-1*β* (inflammasomes: CD206^+^ / CD11c^+^)
	Adipose tissue macrophages (obesity)	IL-6
		Nitric oxide (NO)
		CD11c and immune nitric oxide synthase (iNOS)
		TNF-*α*
		IL-1*β*

Valdearcos et al. [59]; Erta et al. [60]; Franco and Fernández-Suarez [61]	Microglia M2	IL-4R*α*
		IL-10
	Microglia M1	
		IL-1*β*
		IL-2
		IL-6
		IL-12
		IL-15
		IL-17
		IL-18
		IL-23
		IFN-*γ*
		TNF-*α*
		c-Jun N-terminal kinases (JNKs) increased
		Nuclear factor kappa-light-chain-enhancer of activated B cells (NF-kB) increased
		Monocyte chemoattractant protein-1 (MCP1)
		CX3CL1 (fractalkine: chemokine (C-X3-C motif)) (ligand 1)
		Macrophage-stimulating protein receptor (MST1R)

IL: interleukin; TNF: tumor necrosis factor; TGF: tumor growth factor.

**Table 2:** Characteristics and summary results of neuroimaging studies on obesity.

Author, country	Study population	Assessment	Main findings
	*N*; age: mean (SD) years; BMI: mean (SD) (kg/m2)		
Herrmann et al. [72],Germany	Meta-analysis of 10 studies [73-82]	MRI studies	Obese status: ↓ GMV: B IFG (including insula), L MFG, L preFC, L MTG, L PCG, L cerebellum, BA 34 (including L amygdala and L lenticular nucleus)
		Voxel-wise meta-analysis: GMV	
		BMI	

Taki et al. [83], Japan	*N* = 1,428 Men = 690, A: 44.5 (16.1) Women = 738, A: 46.4 (14.1)	MRI: VBM: GMV, GMr	Men: ↑ BMI, ↓ GMV: B uncus, B cerebellum (anterior lobe), L FG, R SPL, R preCG, R IFG, B precuneus, B SFG, R midbrain
		BMI	↑ BMI, ↑ GMV: B IFG, B cerebellum (posterior lobe), R SFG, R STG, R ITG, L MTG, B Th (pulvinar), R CG, B Cn (heads), L preCG
			No significant correlations in women

García-García et al. [86], Canada	Meta-analysis of 21 studies (including 73, 76-80, 82-83, 85, 88, 115) N = 5,882	MRI studies: VBM analyses: GMV	Obesity-related variables are associated with ↓ GMV mainly in mPFC, B cerebellum, L TPole
		Obesity-related variables (BMI, WC, WHR, BFD)	Less robust results: preCG, IPC

Yokum et al. [87], USA	*N* = 83 (women), A: 18.4 (2.8) BMI range: 17.3–38.9	MRI: VBM:	Total GMV: obese < OW, lean
		WMV and GMV	Overall WMV: obese < OW
		BMI	↑ BMI, ↑ GMV: MOG
			↑ BMI, ↑ WMV: MTG, FG, PHipG, RO, DS
			One-year follow-up: ↑ BMI, ↓ GMV: SFG, MF

Papageorgiou et al. [89], Greece	*N* = 268, A: 46.3 (16.1) Obese = 52 OW = 96 Normal weight = 120	MRI: DTI: brain fractional anisotropy	↑ BMI was related with ↓ ATR, PTR, IFOF, ILF, SLF, CC, UF, IC, CST, cingulum
		WM	
		BMI	

Repple et al. [90], Germany	*N* = 1,433 Münster Neuroimaging Cohort = 369 Human Connectome Project = 1064	MRI: DTI: brain fractional anisotropy, WM	↑ BMI globally, ↓ WM integrity (replication in an independent sample)
		BMI	

Kullman et al. [91], Germany/UK	*N* = 48	MRI: DTI, voxel-based quantification analysis	↑ BMI, ↓ predominantly in the SLF, ATR, IC, and CC
	Obese = 12, A: 26.9 (4.5), BMI: 33.2 (3.2)	WM	
	OW = 12, A: 26.1 (2), BMI: 28.1 (1.4)	BMI	
	Lean = 24, A: 26.7 (3.7), BMI: 22.4 (1.6)		

Kim et al. [92], South Korea	*N* = 54 Obese = 27, A: 23.15 (3.2), BMI: 30.12 (3.8); lean = 27, A: 26.1 (5.78), BMI: 21.76 (1.7)	MRI: vertex-based and shape analysis	↑ BMI, ↓ B Cn
		Subcortical structures volume	↑ BMI, ↑ B Th, B Pu, B GP, B hip
		BMI	

Raji et al. [93], USA	*N* = 94	MRI: tensor-based morphometry	Globally: ↑ BMI, ↓ GMV and WMV
	Obese = 14, A: 76.9 (2.8), BMI: 32.9 (2.5)	BMI, FPI, T2DM	↑ BMI, ↓ V: OFC, hip, Pu, GP, Th
	OW = 51, A: 77.2 (2.6), BMI: 27.5 (1.4)		↑ FPI, ↓ V: splenium CC, OFC, hip
	Lean = 29, A:77.5 (4), BMI: 22.5 (1.9)		↑ T2DM, ↓ V: genu and splenium CC, ACC, mTL, OL, Cn, Pu, GP

Dekkers et al. [94], Netherlands	*N* = 12,087 (from UK Biobank study)	MRI: multiparametric brain mapping; DTI	In women: ↑ TBF, ↑ GMV, WMV; ↓ GP
		TBF	In men: ↑ TBF, ↓ GMV, Th, Cn, Pu, GP, hip, Nac

Le et al. [96], USA/Taiwan	*N* = 105 (58 women), A: 31.4 (12.6)	MRI: VBM and rsFC analyses Hypothalamic GMV	↑ BMI, ↑ hypothalamic GMV, and ↑ connectivity with the insula, Th, GP, and cerebellum but ↓ with hypothalamic connectivity with the SPL
	BMI: 25.8 (4.7)	BMI	

Ou et al. [97], USA	*N* = 24	MRI: VBM and TBSS analyses: GMV, WMV	Obese: ↓ GMV: R MTG, B Th, L SPL, L preCG, postCG, L cerebellum
	Obese = 12, A: 9.1 (0.9), BMI: 24.4 (3.4)	BMI	Obese: ↑ WMV: B posterior IFOF, B posterior SFOF, B SCR
	Lean = 12, A: 9.8 (0.7), BMI: 15.8 (1)		

Perlaki et al. [98], Hungary/Netherlands/ Germany/Sweden	*N* = 51, (32 female), A: 13.8 (1.9)	MRI: VBM: GMV, GMM	VBM analysis controlled for age, sex, and ICV; no significant associations between GMM and BMI z-score
		ROIs	ROI: ↑ BMI z-score, ↑ B amygdala, ↑ Nac
		BMI z-score	

Sharkey et al. [100], Canada	*N* = 378 (from NIH Pediatric MRI Data Repository); age range: 4–18	MRI: cortical thickness	No significant correlations were found between cortical thickness and BMI z-score using FDR multiple comparison correction
		ROIs	
		BMI z-score	

Yokum and Stice [101], USA [72]	*N* = 162, (81 female), A: 15.3 (1.1)	MRI: VBM: GMV, WMV	BF gain < BF loss: Pu
	*N* = 60 completed a second scan at two- or three-year follow-up.	BMI: BF gain, BF stability, BF loss	BF gain > BF loss: dorsal/subgenual ACC
			BF gain > BF stability: subgenual ACC

Marqués-Iturria et al. [103], Spain	*N* = 39	MRI: ICV, TGM, CGM, sub-CGM, WMV, mean surface, mean thickness	↑ BMI, ↓ L CGM: SFG, MFG (caudal), ACC (rostral and caudal), preCG
	Obesity = 19, A: 33.7 (5.7), BMI: 36.1 (5.9)	BMI	↑ BMI, ↓ R CGM: mOFC, SFG, MFG (rostral), FPole, OFC
	Lean = 18, A: 32.3 (5.9), BMI: 22.54 (1.94)		↑ BMI, ↓ V: L vDC, R vDC, brainstem

Ottino-González et al. [104], Spain/Canada	*N* = 63	MRI	Overweight: ↑ AL index, ↓ cortical thickness Lean: ↑ AL index, ↑ cortical thickness in pars triangularis, L medial SFG, supramarginal gyrus, IPC, precuneus
	OW-obese = 34, A: 31.8 (6.1), BMI: 31.4 (5)	Cortical thickness	
	Lean = 29, A: 30.07 (6.2), BMI: 22.35 (1.82)	BMI, WC	
		AL index (15 biomarkers)	

Shan et al. [105], China	*N* = 76	MRI: GMV	↑ Creatinine, ↑ GMV ↑ HbA1c, LDL, triglyceride, ↓ GMV
	Obese = 37, A: 27.8 (6.9), BMI: 40 (6.5)	BMI, DBP, creatinine; FBS, HbA1c; HDL, LDL; SBP, SUA, triglyceride	
	Lean = 39, A: 26.7 (6.8), BMI: 21.8 (1.8)		Obese < lean
			R SPG, SOG, AG, cerebellum

Beyer et al. [106], Germany	*N* = 748, A: 68.4 (4.8)	MRI: VBM: GMV, ICV	Metabolic profile (↑ BMI, WHR, HbA1c, leptin, CRP, ↓ adiponectin) associated with ↓ GMV
	BMI: 27.7 (4.1)	BMI, WHR, metabolic profile (adiponectin, CRP, HbA1c, HDL, IL-6, SBP, TC)	Th, B insular Cx, L amygdala-hip, TPole, cerebellum

Kotkowski et al. [107], USA	*N* = 208	MRI: VBM: GMV	Metabolic syndrome was associated with ↓ GMV
	MetS = 104, A: 37.3 (13.2), BMI: 34.5 (5.5)		Cerebellum, OFC, R insula, cuneus, B Cn (body), R TL, R amygdala, BA34.
	Controls = 104, 37.3 (13.2), BMI: 24.3 (3.8)	MetS	

Climie et al. [108], Australia	*N* = 560	MRI: VBM: GMV, hip volume	T2DM is associated with ↓ GMV, ↓ hip volume, ↑ WHR, BMI, ↓ physical activity
	T2DM = 258, A: 67 (7)	WHR, BMI, physical activity (steps/day), and blood biochemistry	T2DM-GMV association is mediated by WHR
	Non-T2DM = 302, A: 72 (7)		

Nouwen et al. [109], UK	*N* = 53	MRI: VBM, TBSS: GMV, WMV, T2DM, obesity GMV: control > T2DM: B Cn, B Pu	Control > obese: R hip, R amygdala-hip, L amygdala, L Pu
	T2DM = 14, A: 16.1 (1.5)		Control > T2DM + obese: R Pu/Cn, L Cn, L Pu, L Th/Cn, L hip/amygdala
	Obesity = 20, A: 14.9 (2)		WM: T2DM < controls: L CST, mCC, L fornix, L ThR, L RLIC, L IFOF, R ACR, CC (genu), L uncinate, cingulum

Bernardes et al. [110], Brazil/ Netherlands	*N* = 75	MRI: CT, surface area, GMV	CT: obese + T2DM < controls, obese (normoglycemic) < controls: R insula
	Obese + T2DM = 28, A: 60.4 (5)	T2DM, adiposity, glucose, and insulin	Surface area: obese + T2DM < controls: L lateral occipital
	Obese (normoglycemic) = 16, A: 58 (8.4)		Surface area, GMV: Th, obese + T2DM < obese (normoglycemic): L paracentral
	Control = 31, A: 57 (7.1)		

Rofey et al. [111], USA	*N* = 15	MRI: GMV	GMV, WM integrity: Cn, Th
	T2DM = 5, A: 18 (1.4)	BMI, T2DM	T2DM < obese < lean: Cn
	Obese (without T2DM) = 5, A: 15.4 (2.2)		T2DM < obese; T2DM < lean: Th
	Lean = 5, A: 14.5 (2.5)		

Redel et al. [112], USA	*N* = 40	MRI: VBM: GMV, voxel-wise GM, WMV	Global GMV: obese + T2DM < control
	T2DM = 20, A: 16.7 (2.6), BMI: 37.8 (4.7)	BMI, T2DM	Regional GMV: Obese + T2DM < control: R mTL, L fusiform, L IPG, R cerebellum, B ITG, R cingulum, R MOG, R IOG, L Cn, L angular, R lingual
	Control = 20, A: 16.7 (2), BMI: 24.5 (5.2)		Obese + T2DM > control: B Pu, R ITG, R Th, B paracentral

Yoon et al. [113], South Korea	*N* = 150	MRI: GMV, CT	Global mean CT: T2DM + OW and T2DM < control
	T2DM + OW = 50, A: 49 (7.4), BMI: 28.1 (2)	T2DM, BMI	Regional CT: T2DM + OW and T2DM < control: B FG, L ITG, B STG, B SFG, L MFG, B IFC, L insula, B lingual Cx, R OFC
	T2DM = 50, A: 49.3 (8.1), BMI: 22.8 (2)		WM: T2DM + OW and T2DM < control: Fornix, CC, CST, IFOF, ATR
	Control = 50, A: 49 (7.8), BMI: 22.7 (1.8)		Duration-related alteration in CT: T2DM + OW > T2DM

Caunca et al. [114], USA/Japan	*N* = 1,289 (from the Northern Manhattan MRI Substudy), A: 64 (8)	MRI: ICV, mean CT, WMHV	↑ BMI and ↑ WC z-score, ↓ mean CT
			Obese < normal: CT
		BMI z-score, WHR z-score, WC z-score, adiponectin	No association between obesity and cerebral small vessel disease (WMHV)

Hayakawa et al. [116], Japan	*N* = 792	MRI: voxel-wise analyses	Men: ↑ WC, BMI, ↓ GMV: B ITG, R MTG, L fusiform, L Th, L red nucleus, L mFG, R SFG, R IFG, R preCG, R postCG, B cerebellum
	Men = 523, A: 55.3 (9.7)	GMV; GMr, ICV	Women: ↑ WC, BMI, ↓ GMV: B Th, R red nucleus, L preCG, L IFG, L mFG, R hip
	BMI: 24.7 (3.1), WC (cm): 88.5 (8.1)	BMI, WC	Regions were more widespread in men than in women
	Women = 269, A: 55.2 (9.9)		
	BMI: 22 (3.3), WC (cm): 81.2 (9.8)		

Hamer and Batty [117], UK	*N* = 9,652 (from UK Biobank study) Obese = 1,805, A: 55 (7.3)	MRI: GMV	↑ BMI, WHR, total fat mass, ↓ GMV
	OW = 4,167, A: 56 (7.5)	BMI, WHR, total FM, FM index (total FM (kg)/height (m))	Combination of overall obesity (BMI) and central obesity (WHR) was associated with the lowest GMV
	Lean = 3,680, A: 54.7 (7.5)		↑ BMI, ↓ Pu, Pa, Nac
			↑ WHR, ↓ Th, Cn, Pa
			↑ Fat index, ↓ Pu, Pa, Nac

Croll et al. [118], Netherlands	*N* = 3,648 (from Rotterdam Study) Cross-sectional study = 3,648, A: 65.9 (11.1)	MRI: GMV, WMV	↑ BMI and FM index were cross-sectionally associated with ↓ WMV
	Longitudinal study = 1,844, A: 60.9 (9.9)	BMI, DXA: FM index, FFM index	↑ FFM index was associated with ↑ GMV

*Note. Brain Anatomy.* ACC: anterior cingulate cortex; ACR: anterior corona radiata; AG: angular gyrus; ATR: anterior thalamic radiation; B: bilateral; Cn: caudate nucleus; CC: corpus callosum; CG: cingulate gyrus; CST: corticospinal tract; DS: dorsal striatum; FG: fusiform gyrus; FPole: frontal pole; Hip: hippocampus; IC: internal capsule; IFC: inferior frontal gyrus; IFOF: inferior fronto-occipital fasciculus; ILF: inferior longitudinal fasciculus; IOG: inferior occipital gyrus; IPG: inferior parietal gyrus; ITG: inferior temporal gyrus; L: left; mCC: medial corpus callosum; mTL: medial temporal lobe; mOFC: medial orbitofrontal cortex; mFG: medial frontal gyrus; MFG: middle frontal gyrus; mPFC: medial prefrontal cortex; MTG: middle temporal gyrus; MOG: middle occipital gyrus; Nac: nucleus accumbens; OFC: orbitofrontal cortex; OL: occipital lobe; Pa: pallidum; PHipG: parahippocampal gyrus; PTR: posterior thalamic radiation; postCG: postcentral gryrus; pre CG: precentral gyrus; Pu: putamen; R: right; RLIC: retrolenticular internal capsule; RO: Rolandic operculum; SCR: superior corona radiata; SFG: superior frontal gyrus; SFOF: superior fronto-occipital fasciculus; SLF: superior longitudinal fasciculus; SPL: superior parietal lobe; SPG: superior parietal gyrus; SOG: superior occipital gyrus; STG: superior temporal gyrus; Th: thalamus; TL: temporal lobe; TPole: temporal pole; UF: uncinate fasciculus; vDC: ventral diencephalon. *Neuroimaging*. CGM: cortical gray matter; CT: cortical thickness; DTI: diffusion tensor imaging; FDR: false discovery rate; GMM: gray matter mass; GMr: gray matter ratio (percentage of GMV in the intracranial volume); GMV: gray matter volume; ICV: intracranial volume; MRI: magnetic resonance imaging; rsFC: resting-state functional connectivity; ROI: region of interest; sub-CGM: subcortical gray matter; VBM: voxel-based morphometry; V: volume; TBSS: track-based spatial statistic; TGM; total gray matter. *Obesity Assessment.* BMI: body mass index; BMI z-score: standardized BMI; BF: body fat; BFD: body fat distribution; DBP: diastolic blood pressure; DXA: dual-energy X-ray absorptiometry; FFM: fat-free mass; FBS: fasting blood sugar (fasting glucose); FM: fat mass; FPI: fasting plasma insulin levels; HbA1c: glycated hemoglobin; HDL: high-density lipoprotein; LDL: low-density lipoprotein; MetS: metabolic syndrome; OW: overweight; SBP: systolic blood pressure; SUA: serum uric acid; TBF: total body fat; TC: total cholesterol; WC: waist circumference; WHR: waist-to-hip ratio; WMV: white matter volume.
